# Predictors of Changes in Travel Behavior during the COVID-19 Pandemic: The Role of Tourists’ Personalities

**DOI:** 10.3390/ijerph182111169

**Published:** 2021-10-24

**Authors:** Cezar Morar, Alexandru Tiba, Biljana Basarin, Miroslav Vujičić, Aleksandar Valjarević, Liudmyla Niemets, Alena Gessert, Tamara Jovanovic, Marius Drugas, Vasile Grama, Marius Stupariu, Alina Stoica, Tin Lukić

**Affiliations:** 1 Department of Geography, Tourism and Territorial Planning, University of Oradea, 410087 Oradea, Romania; cezar.morar@gmail.com (C.M.); vasile.grama2014@gmail.com (V.G.); marius_stupariu@yahoo.co.uk (M.S.); 2 Department of Psychology, University of Oradea, 410087 Oradea, Romania; mariusdrugas@gmail.com; 3 Department of Geography, Tourism and Hotel Management, Faculty of Sciences, University of Novi Sad, 21000 Novi Sad, Serbia; biljana.basarin@gmail.com (B.B.); vujicicm@gmail.com (M.V.); tamara.jovanovic@dgt.uns.ac.rs (T.J.); lukic021@gmail.com (T.L.); 4 Faculty of Geography, University of Belgrade, 11000 Belgrade, Serbia; aleksandar.valjarevic@gef.bg.ac.rs; 5 Department of Human Geography and Regional Studies, V.N. Karazin Kharkiv National University, 61022 Kharkiv, Ukraine; ludmila.nemets@karazin.ua; 6 Physical Geography Department in Geography Institute, 040 01 Košice, Slovakia; alena.gessert@upjs.sk; 7 Department of International Relations and European Studies, Faculty of History, International Relations, Political and Communication Sciences, University of Oradea, 410087 Oradea, Romania; stoicaalina79@yahoo.com

**Keywords:** SARS-CoV-2, COVID-19, personality, coping, fear of travel, travel behavior

## Abstract

This study investigates travel behavior and psychosocial factors that influence it during the COVID-19 pandemic. In a cross-sectional study, using an online survey, we examined changes in travel behavior and preferences after lifting travel restrictions, and how these changes were influenced by exposure to COVID-19, COVID-19 travel-related risk and severity, personality, fear of travel, coping, and self-efficacy appraisals in the Romanian population. Our results showed that participants traveled less in the pandemic year than the year before—especially group and foreign travel—yet more participants reported individual traveling in their home county during the pandemic period. Distinct types of exposure to COVID-19 risk, as well as cognitive and affective factors, were related to travel behavior and preferences. However, fun-seeking personality was the only major predictor of travel intention, while fear of travel was the only predictor of travel avoidance. Instead, people traveled more cautiously when they perceived more risk of infection at the destination, and had higher levels of fear of travel, but also a high sense of efficacy in controlling the infection and problem-solving capacity. The results suggest that specific information about COVID-19, coping mechanisms, fear of travel, and neuropsychological personality traits may affect travel behavior in the pandemic period.

## 1. Introduction

Life on Earth represents a chain of 4 billion years of fragile and interconnected evolutionary processes. Humans are part of this miracle, and travel is an intrinsic part of the lengthy process of human evolution. Travel shaped the land and changed the Earth’s living sculpture, constituting a stepping stone of humankind’s ingenuity and continuous adaptation to newer and further natural environments. Travel is about progress and civilization; since the beginning, it was part of human nature, as the instinct of preservation or curiosity was the main prehistoric travel motivation. Traders, explorers, scientists, and cultured people traveled further, conquering more and more space and opening new horizons. The influx of Greek travelers to holy places such as Dodona or Delphi led to the extension of Greek culture throughout the Mediterranean Basin. In ancient Rome, the famous bathing facilities thermae and balnea attracted visitors from distant regions. In the Middle Ages, people traveled for mystical reasons, such as priests for religious purposes, or pilgrims visiting holy places. In the following centuries, the revival of trade led to travel for commercial purposes—for instance, to Italian cities, such as Venice, Pisa, Genoa, and other cities of Lombardy and Tuscany. The Renaissance’s academic and artistic focus brought other motivations for travel, as the Grand Tours offered young British aristocrats opportunities to discover Europe, and to broaden their horizons through new experiences and knowledge. Only two centuries ago, the rural societies in Europe met a turning point in history—the Industrial Revolution, whose innovations led to a strong development of the means of transport and communication, in addition to great improvements in living standards that generated more financial and temporal resources. People would travel to escape the polluted and crowded industrial cities, heading to coastal areas or pristine mountain environments for leisure and recreation. In the past century, despite wars, economic recessions, geopolitical changes [1,2,3], and terrorist attacks, tourism has become one of the most dynamic sectors of the global economy. A combination of desire, mobility, and accessibility made possible the existence of mass tourism. Travel came to involve millions of people heading to new tourist destinations and seeking new experiences in distant environments. Tourism enabled the development of a new economic sector—the provision of services—while international tourism became the most important element of international trade budgets.

In this fertile context, international tourist arrivals exploded, from just 25 million in 1950, to 1.5 billion in 2019 (+ 4% compared to the previous year) (https://www.unwto.org/international-tourism-growth-continues-to-outpace-the-economy, 8 October 2021) [4]. According to the same international body (UNWTO), in the same year (2019), tourism became the third largest export category, growing faster than the world economy. Then, in early 2020, SARS-CoV-2 almost stopped the world. The impact on tourism was devastating (USD 2 trillion losses in global GDP, USD 1.3 trillion losses in tourism receipts) (https://www.unwto.org/covid-19-and-tourism-2020, 8 October 2021). Tourism registered an unprecedented fall of −73.9% in 2020, bringing the sector close to its 1990s level (SARS (2003) generated a fall in international tourism of −0.4%, while the global economic crisis (2009) caused a fall of −4%)(https://www.unwto.org/covid-19-and-tourism-2020, 8 October 2021). This plunge continued in 2021, with the data showing a decline of 87% in international arrivals in January 2021, compared to January 2020 (https://www.unwto.org/taxonomy/term/347, 8 October 2021).

The WHO initially declared the outbreak a global health emergency, and then a SARS-CoV-2 pandemic. As a result, travel restrictions were imposed, affecting 100% of worldwide destinations, while 27% of all destinations worldwide completely closed their borders (https://www.unwto.org/covid-19-and-tourism-2020, 8 October 2021). In this context, the issue of restarting tourism has become more important than ever. At the same time, understanding the travel behaviors in the complicated context of COVID-19 is of great importance [5].

In an effort to control the spread of the virus, public authorities implemented policy-level actions and strategies (closing schools, online teaching, home working, closing stores and restaurants, etc.) [6]. As the people’s mobility was spreading the virus, restraining mobility was also a key mitigation policy, so measures included closing international borders, closing airports, limiting community contacts, and restricting international travel as mitigation policies [6,7,8]. In addition, in an effort to limit travel, social restriction measures were imposed on public meetings, social, sporting, and cultural events, and public transportation or taxi operations [7,8]. The imposed mobility restrictions were implemented in accordance with different local cultures, administrative organizations, and socioeconomic conditions [6]. These mobility restrictions were applied at different administrative levels: international (e.g., closing countries), national (e.g., closing regions and cities), regional (e.g., city lockdowns), and local (e.g., restraining walking or motorized transportation, closing public places) [9]. The restrictions seriously affected the diverse travel needs (tourism, working, shopping, etc.), which further generated changes in travel behaviors.

Especially for tourism, the fear of infection strongly influences travel behaviors and daily travel activities (e.g., shopping routines, recreational activities, heritage explorations) [10,11,12,13,14,15,16,17]; concerns about infection rates and sociodemographic characteristics have been raised as factors influencing travel satisfaction [2,18]. Studies show that cases and deaths in the COVID-19 context are lower in countries where people travel less to other countries [8,19,20], while unrestrained mobility significantly accelerates the spread of COVID-19 [21]. In contrast to the massive plunge in the travel sector, both the intensity and the average duration of cycling trips have greatly increased during the pandemic (https://www.tandfonline.com/doi/pdf/10.1080/13683500.2020.1798895?needAccess=true, 8 October 2021) [22,23]. The behavioral changes during the pandemic have affected the use of public transportation (e.g., buses, subways), as the sector has seen a strong downturn (in New York City, trips were down by 94% between April 2019 and April 2020) [24,25,26]. The aviation industry also took a hard hit, with an overall loss of 2699 million passengers (−60%) in 2020 compared to 2019 (and an approximately USD 371 billion loss of gross passenger operating revenues) (https://www.icao.int/sustainability/Documents/COVID-19/ICAO_Coronavirus_Econ_Impact.pdf, 8 October 2021).

The coronavirus pandemic has had a great impact on our daily lifestyles, bringing significant changes to millions of people. Changing individual behavior is a strong mitigating and adapting factor [27]. Knowing more about changes in travel behavior and changes in preferences during the COVID-19 pandemic is useful for tourism operation planning and policymaking, not only for this pandemic, but also for future events of this kind. The planning of smart measures and policies are great tasks for decision makers in the public sectors, or for private travel service providers, for better planning their operations. Policies to promote travel during the COVID-19 pandemic would benefit a lot from knowledge regarding personal factors that influence travel behavior during pandemics. The knowledge that perceptions of risk have a larger influence than coping with COVID-19 can suggest different strategies to increase travel. The knowledge that perception of risk to loved ones rather than the self influences preferences for specific types of travel calls for promotion strategies focused on taking care of loved ones during travel. Knowing that these factors preferentially affect particular types of travel would stimulate a focus on using targeted strategies to promote particular types of travel. Moreover, these factors may function as tags for applying different strategies. For instance, knowing that persons who have high levels of fear of infection during travel will be spared if they use disengagement coping, or will perceive an increase in self-efficacy for preventing infection via personal behavior, suggests promotion strategies for “fearful travelers” focused on increasing self-efficacy for specific types of travel. Since little research has examined individuals’ psychological responses and coping mechanisms in a pandemic travel context, we aim to investigate individual psychological and personality variables involved in travel behavior, preferences, and responses to the COVID-19 pandemic.

### 1.1. Literature Review

#### 1.1.1. Risk Perception and Disease Severity

Perceiving a high probability of contracting a severe disease is the main factor that leads to cancelation of visits and travel avoidance [28]. The higher the threat level in a destination, the lower the intent to travel. Risk or threat susceptibility refers to subjective estimations of the probability of contracting a disease or a virus. Threat severity refers to subjective estimations of the severity of the consequences or seriousness of the threat [29]. Risk and severity estimations that influence travel are related to objective threat exposure and demographic characteristics such as gender [30], age [31,32], and nationality [33]. Additionally, several subjective factors have been shown to result in exaggerated threat perceptions, such as travel experience [34], personality [30,34], and memory accessibility [30,33]. Similarly, the intention to travel during COVID-19 has been shown to be influenced by the perceived risk and severity of SARS-CoV-2 infection across the world in tourists from Germany, Austria, and Switzerland [35], China [36], Greece [37], Bulgaria [38], and Serbia [39,40]. However, little is known about how different types of exposure to the threat of SARS-CoV-2 (home exposure, media exposure, threat levels at the destination of travel) and different types of risks at travel destinations (to the self or to others) predict changes in travel behavior. Furthermore, we do not know how COVID-19 exposure- and risk-related predictors interact with psychological factors (e.g., self-efficacy in controlling the disease, positive growth beliefs about COVID-19, coping, fear of travel, and personality) in explaining changes in travel behavior.

#### 1.1.2. Tourists’ Fear of the COVID-19 Pandemic 

Given that the COVID-19 pandemic is a major threat to health, survival fear was one of the first natural reactions to it [41]. Several studies point to the fact that fear, anxiety, and mental health problems were increased during the pandemic recovery period [42]. Other studies suggest that fear of travel during the pandemic is the main predictor mediating the impact of other factors on travel behaviors [43]. Furthermore, different reactions to manage fear of travel have been identified, such as cautious travel, travel in groups or with friends, pre-travel preparation, and choosing familiar places [44]. Although it is known that the level of fear experienced by individuals largely depends on their specific personality type, little is known about how tourists experience and react to fear of travel depending on their personality type.

#### 1.1.3. Resilience Factors: Coping, Self-Efficacy, and Positive Growth

Resilience refers to individuals’ capacity to cope with a stressor, to return to pre-stressor status quickly, and/or to grow and positively adapt after a crisis. Different factors have been proposed as affecting resilience, such as (1) biological traits, (2) general beliefs about stress, (3) coping mechanisms, (4) personality factors, and (5) positive emotions. Individuals have ways of dealing with threat or anxiety about SARS-CoV-2 infection that reduce its impact and promote safety. Knowing how to avoid SARS-CoV-2 infection and its severity, along with trusting in the success of these methods, may reduce the perceptions of threat and COVID-19-related anxiety [43]. Previous studies showed that the most efficient coping mechanisms for controlling SARS-CoV-2 infection and reducing fear of COVID-19—such as problem-solving, self-supported emotional coping, social-supported emotional coping, and detachment—enhance resilience and the intention to travel [43]. Furthermore, self-efficacy (how much an individual believes they have the means to prevent being infected) and response efficacy (how much an individual believes that what they do to control the infection is efficient) for controlling SARS-CoV-2 infection have also been shown to promote travel [43]. Although previous studies showed that positive beliefs, increased self-efficacy in controlling SARS-CoV-2 infection, and coping mechanisms may promote travel [43], no studies investigated how such beliefs are related to changes in preferences related to travel during the COVID-19 pandemic.

#### 1.1.4. Personality and Travel

It is well recognized that personality plays a special role in both perceptions of risks and preferences related to travel. Studies have shown that individuals with dysfunctional personality traits have more negative responses to pandemics. For instance, individuals with negative affectivity, detachment, antagonism, and psychoticism had more emotional problems during the first month of the COVID-19 pandemic [45]. Those with higher levels of neuroticism estimated longer COVID-19 pandemic duration [46], and those with high levels of depressive, cyclothymic, and anxious affective temperaments [47] and neuroticism [48] had more psychological distress. Conversely, higher levels of resilience-type personality traits seem to be protective against negative effects of the pandemic. For instance, individuals with high levels of emotional intelligence displayed efficient coping mechanisms and resilience [49].

Little is known about how personality affects travel intention and preferences during health crises such as the COVID-19 pandemic. Previous studies have shown that individuals who display higher levels of consciousness and novelty-seeking show more cautious behavior in traveling conditions, while individuals with higher levels of openness show less cautious behavior as a response to risk [50]. Other studies found most relations between personality traits and reactions to COVID-19 to be of small magnitudes [45]. Although biology-related personality traits may be more important for our reactions to threats such as SARS-CoV-2 infection, little research has investigated biological personality traits in relation to travel during the COVID-19 pandemic. As an exception, Oniszczenko (2021) found that behavioral inhibition predicts COVID-19-related fear for the health of loved ones in women, but he did not investigate behavioral inhibition in relation to travel [51].

A well-recognized neuropsychological personality model is the BIS/BAS model. The BIS/BAS model is based on Gray’s (1982) reinforcement sensitivity theory (RST). According to this model, personality includes two basic brain-motivational systems responding to appetitive or aversive stimuli: the behavioral inhibition system (BIS), and the behavioral activation system (BAS). The BAS has three subsystems, each with a distinct specialized function. BAS reward responsiveness is involved in controlling the positive responses to rewards, BAS drive in the persistence of achievement of goals, and BAS fun-seeking in the desire for new stimuli and impulsivity [52]. The BIS is responsible for responses to conditioned aversive stimuli (e.g., punishment signals; [53]) and susceptibility to negative feelings such as fear, anxiety, frustration, or sadness in response to negative signals (e.g., punishment, lack of reward, novelty) [54]. Traveling under threats such as infection with COVID-19 during the pandemic may be under the regulation of the BIS, with individuals with more sensitive BISs being prone to avoidance of travel. On the other hand, individuals with a sensitive BAS may be prone to taking risks and engaging in travel under such risks, while high levels of both BIS and BAS may promote increased cautious travel.

In sum, during the COVID-19 outbreak, tourism has been significantly limited due imposed government restrictions, with travel being considered a high-risk activity. Moreover, significant reductions in travel behaviors have arisen from (1) exposure to COVID-19, (2) social-media-promoted uncertainty and negativity about the pandemic, and (3) individual psychological reactions to the pandemic. Little is known about personal factors that can be targeted to boost travel and improve post-pandemic economic recovery. Several studies revealed that tourists’ travel behaviors during pandemics may be influenced by their risk perceptions (e.g., [19,55], cognitive [43] and affective responses (e.g., fear of travel, [43])), coping responses, and motivations (e.g., [56]). However, studies have not investigated reported changes in preferences during travel, and how they relate to cognitive, affective, and neuropsychological personality factors. In our previous papers we have considered an integral assessment of ethnic tourism in Ukraine [57], the current state of child and youth tourism development in Ukraine [58], perspectives of tourism development in terms of the water crisis in Iran [59], features of pilgrimage [60], and rural tourism in Ukraine [61]. Adding an investigation of both external and internal factors that affect tourism under crises such as the COVID-19 pandemic will add significantly to our previous efforts.

### 1.2. The Study

Although vital for reinvigorating tourism during and after the COVID-19 pandemic, little is known about how various types of exposure to COVID-19, risk perceptions, psychological reactions, and neuropsychological personality traits predict travel behavior and preferences during pandemics. To fill this knowledge gap, this study aims to explore self-reported changes in travel behaviors and preferences in relation to psychological reactions to the COVID-19 pandemic and neuropsychological personality traits. The key questions underpinning the research are:

(1) How have travel behavior and preferences changed during the SARS-CoV-2 pandemic in our sample?

(2) What are the processes that predict travel intention, avoidance, and cautious travel during the pandemic?

(3) What are the environmental, travel-related health risks, personality, affective, cognitive, and emotional factors that predict preferences for safety, stimulation, prestige, and relief?

## 2. Materials and Methods

### 2.1. Construct Measures

A cross-sectional design was used to test the hypotheses. Several scales and items adapted from previous relevant studies were administered to ensure content validity. Two translators translated the English instruments into Romanian independently.

#### 2.1.1. Demographic Information

As demographic variables, respondents were asked to indicate their gender (“male”, “female”), their age (in decade categories), their highest educational level obtained (from ”primary school degree” to “postgraduate”), employee status (“employed”, “not-employed”, “student”, “self-employed”, “unemployed due to COVID-19 crisis”, “retired”), whether they had already been infected with the virus (“yes”, “no”), and whether or not they were vaccinated.

#### 2.1.2. Previous Exposure to COVID-19

Three items measured media exposure (“How frequently have you watched or used media material related to COVID-19?” (very frequent to none), exposure status (yes/no), infection status (yes/no), and impact of COVID-19 (no impact, little impact, high impact, very high impact)).

#### 2.1.3. Exposure to COVID-19 and Risk at Travel Destination

A 7-point ordinal scale by Georgiou et al. (2020) was adapted to travel destinations, with the following answers: 1 = unaware of any COVID-19 in the destination country; 2 = COVID-19 in the destination country, 3 = COVID-19 in the destination city; 4 = COVID-19 in the local destination area; 5 = person(s) you know affected in the travel destination; 6= someone close to you infected in the travel destination; 7 = currently or have been affected by COVID-19 in the travel destination. Participants also rated how at risk they were of contracting SARS-CoV-2 at their travel destination (1 = very low; 2 = low; 3 = moderate; 4 = high; 5 = very high), of transmitting the virus to people who were close to them (1 = very low; 2 = low; 3 = moderate; 4 = high; 5 = very high), and the severity of the disease contracted at the travel destination (1 = no severity; 2 = little severity; 3 = moderate; 4 = severe; 5 = very severe) [62].

#### 2.1.4. The Pandemic (COVID-19) Anxiety Travel Scale (PATS)

The PATS [63] is a 5-item scale that measures the level of pandemic-induced anxiety. Participants indicate their feelings and thoughts when thinking about travelling during the pandemic. Respondents were asked to rate their level of anxiety using a 5-point Likert-type scale (from 1 = not at all, to 5 = very much). The internal consistency of the scale was good in the current sample (Cronbach’s alpha = 0.87).

#### 2.1.5. BIS/BAS Scale

The BIS/BAS scale [52] is a 20-item self-report measure that assesses trait sensitivity levels of the behavioral activation system and the behavioral inhibition system. Likert-type response scales ranging from l (very true for me) to 4 (very false for me) were used. The BIS scale consists of seven items, two of which have reversed scoring and four filler items. Two global scores are drawn: global BAS and BIS scores. The BAS scale includes three separate subscales: (1) BAS reward responsiveness (5 items), (2) BAS drive (4 items), and (3) BAS fun-seeking (4 items). The internal consistency of the scale was acceptable in the current sample (Cronbach’s alpha = 0.73).

#### 2.1.6. The Coping Scale

Items adapted from the Brief-COPE [64] and used in previous studies regarding adaptation to COVID-19 and travel [43] were included. Items referred to problem-focused (e.g., “I tried to develop a strategy about what to do about COVID-19”, 3 items, Cronbach’s alpha = 0.87), self-supported emotional (e.g., “I learned to live with COVID-19”, 4 items, Cronbach’s alpha = 0.76), social-supported emotional (e.g., “I got emotional support from others regarding COVID-19”, 4 items, Cronbach’s alpha = 0.85), disengagement (e.g., “I refused to believe that COVID-19 happened for real” 4 items, Cronbach’s alpha = 0.76) [43], and collective coping (e.g., “I used social media to find support to face COVID-19 crisis together with others” 3 items, Cronbach’s alpha = 0.79) factors.

#### 2.1.7. The Short Form of the Posttraumatic Growth Inventory (PTGI-SF)

The PTGI-SF is a 10-item self-report measure that assesses positive adaptation to crises. Items reflect positive consequences of the COVID-19 crisis in areas of personal strength, relating to others, new possibilities, spiritual change, and appreciation of life (e.g., “I discovered that I’m stronger than I thought I was”). Instructions were adapted to refer to the COVID-19 crisis. Participants endorsed their response on a six-point scale from 0 (0 = I did not experience this change as a result of the COVID-19 crisis) to 5 (5 = I experienced this change to a very great degree as a result of the COVID-19 crisis). The internal consistency of the scale was good in our sample (Cronbach’s alpha = 0.96) [65].

#### 2.1.8. Self-Efficacy for Controlling SARS-CoV-2 Infection

A four-item scale measuring self-efficacy based on previous works [18,66], adapted to controlling COVID-19 infection in relation to travel [43], was used in this study. The internal consistency of the scale was acceptable in our sample (Cronbach’s alpha = 0.72).

#### 2.1.9. Travel-Related Measures

We used several measures for travel-related variables: intention to travel (three items, [67], Cronbach’s alpha = 0.88), avoidance of travel (two items, [43], Cronbach’s alpha = 0.89), and cautious travel (three items, [43], 2021, Cronbach’s alpha = 0.88). Additionally, the respondents were asked to indicate their travel choices in terms of type and location. Preferences for the years before and after the outbreak of the pandemic were rated on a 1–5 Likert scale (1 = not important, 5 = very important) for new knowledge (2 items, Cronbach’s alpha = 0.73), stimulation (3 items, Cronbach’s alpha = 0.92), prestige (4 items, Cronbach’s alpha = 0.89), relief (5 items, Cronbach’s alpha = 0.85), and health (6 items, Cronbach’s alpha = 0.92)

### 2.2. Data Collection

An online survey was conducted among Romanian residents who had travelled within the past 12 months and experienced the outbreak of COVID-19 within Romania. The survey was generated via Google Forms and shared on local social media groups and by email to individuals in Bihor County, a region in West Romania and Oradea (the main city of the county). The study was conducted from May to October 2021, with data collection starting approximately one year and two months after quarantine restrictions in Romania, and ending before the fourth wave of COVID-19 infections in Romania. After eliminating outliers and bad responses (fast responses and pattern answers), 110 responses were retained for final analysis. A pilot test of the online survey was conducted to check the appropriateness of the survey. After giving informed consent, the participants completed the survey. The study was conducted in accordance with the guidelines of the Declaration of Helsinki, and approved by the Ethics Committee of the University of Oradea.

### 2.3. Data Analysis

The statistical model involved independent Student’s *t*-tests for comparing the group differences in travel behavior between vaccinated and non-vaccinated participants. Paired *t*-tests were used to compare significant changes in travel preferences from the year before the pandemic. Significant differences were used as dependent variables for regression models. To analyze the correlations, we conducted a multivariate regression analysis to correct for multiple analyses. For personality scores, an expectation-maximization algorithm was used to handle the missing data. t-tests were used to analyze changes in preferences. For correlational and regression analyses, outlying data (above 3 standard deviations) were removed based on IBM SPSS Statistics for Windows, Version 23.0, IBM Corp, Armonk, NY, USA. The regression models were checked for multicollinearity. All values of the variance inflation factor (VIF) were below the maximum threshold level of 10. We conducted a series of stepwise regressions to predict travel behaviors. The procedure was repeated separately for each travel behavior: intention to travel, cautious travel, and travel avoidance.

## 3. Results

### 3.1. Demographics

Demographics Are Described in Table 1 Below.

### 3.2. Home Exposure and Estimations of Risk of SARS-CoV-2 Infection at Travel Destination (Research Question 1)

Descriptive analyses showed that 71.8% of the Romanian respondents planned to travel after the removal of restrictions. Among the respondents, 44.5% intended to travel in places where they knew that SARS-CoV-2 was present in the destination country, city (17.3%), or local area (20%), though only 1.8% to places where they knew persons who were infected in that destination. When asked about their intention to travel (e.g., to rate how much they agree that they will travel any time they have the chance after the removal of restrictions), 68.1% of the participants agreed that they would travel, with only 22.7% being neutral about this statement, and only 9% disagreeing. *When asked about* whether they would avoid travel as long as COVID-19 still existed (travel avoidance) 27.2% of participants stated that they would avoid travel, 17.2% were neutral about whether they would avoid travel, and 55.4% disagreed that they would avoid travel. When asked if they would check during their travels for the necessary protections to prevent being infected, 65.4% of respondents agreed with the statement, 20% were neutral, and 14.5% of respondents disagreed.

The analyses of the perceived risk or probability of being infected in the travel destination showed that few respondents planned to travel to destinations where they perceived a high risk (4.5%) or very high risk of being infected (0.9%) at the destination. However, around half of respondents (41.8%) planned to travel to destinations where they perceived a moderate risk, 31.8% of low risk, and 20.9% of very low risk of being infected when traveling to that destination.

The analyses of the estimated severity of being infected in the travel destination showed that few respondents planned to travel to destinations where they perceive infection as being very severe (5.5%), or severe (9.1%). However, 28.2% planned to travel to destinations where they estimated is the severity of infection as quite severe, and 49.1% to destinations where they estimated infection as having a low severity. Only 8.2% of respondents estimated the infection at their intended destinations as having no severity. Regarding the perceived risk of transmitting the infection to other close people if they become infected at the travel destination, 15.5% of respondents planned to travel to destinations where they perceived a very low risk, 27.3% low risk, 30% moderate risk, 19.1% high risk, and 8.2% very high risk. We asked the participants to rate the importance of different characteristics of travel, both at the moment of responding to the questionnaire and before the year of the pandemic. At the moment of the responding to the questionnaire, the most important travel preferences were to be with family and friends (M = 4.26, SD = 1.01), relaxing and enjoying free time (M = 4.20, SD = 1.04), keeping healthy (M = 4.18, SD = 1.06), hygiene (M = 4.14, SD = 1.07), to be physically active (M = 4.05, SD = 1.10), quiet area (M = 3.93, SD = 1.15), high quality of facilities (M = 3.97, SD = 1.17), no queues for services (M = 3.86, SD = 1.16), absence of agglomeration (M = 3.85, SD =1.10), and quality of the environment (M = 3.84, SD = 1.19).

Participants endorsed the 10 most important travel preferences for the year before the pandemic as being relaxing and enjoying free time (M = 4.31, SD = 0.98), to be with family and friends (M = 4.23, SD = 1.05), hygiene (M = 4.16, SD = 1.07), keeping healthy (M = 4.15, SD = 1.06), quiet area (M = 4.04, SD = 1.13), to be physically active (M = 4.00, SD = 1.10), clean and comfortable accommodation (M = 3.98, SD = 1.15), absence of agglomeration (M = 3.87, SD = 1.11), no queues for services(M = 3.80, SD = 1.19), and entertainment and fun (M = 3.75, SD = 1.22).

In our sample, in the year of the pandemic, preferences for high quality of facilities entered the top 10, replacing preferences for entertainment and fun that were present in the top 10 of the year before the pandemic.

### 3.3. Differences in Travel Types and Preferences (Research Question 1)

Given previous proposals regarding changes in travel during health crises [45], we hypothesized that Romanian participants would report a tendency to prefer traveling near home and by car, as opposed to destinations abroad and in groups, when compared with previous destinations reported in the year before the pandemic. Furthermore, we expected an increase in preferences for travel based on motivations for safety, and to reduce stress and depression. We asked participants to report how often they traveled the year before the pandemic and in the year of the pandemic using four categories: not at all, 1–3 times, 4–7 times, and more than 10 times. McNemar’s test was used to compare the differences in travel in the years before and during the pandemic. Analyses showed that significantly fewer participants reported having 1–3 trips abroad in the year of the pandemic (*p* < 0.001) than they had the year before. Additionally, more participants reported that they did not travel abroad during the pandemic than participants that reported that they did not travel the year before (*p* < 0.001). A similar pattern was observed regarding group travel in the home country. A different pattern emerged for personal and family travel in the local county and surroundings. While more participants reported that they traveled 1–3 times during the pandemic than the year before (*p* = 0.029), fewer participants reported they traveled more than 10 times (*p* = 0.021) in their county during the pandemic than the year before. Thus, participants who traveled more in their home county before the pandemic (“high travelers”) reported traveling less often during the pandemic. A similar trend was found for traveling in the home country, but did not reach statistical significance.

We asked participants to rate how important different preferences are when they travel, referring to both the year before COVID-19 and in the context of the continued presence of the SARS-CoV-2 virus. Paired sample t-tests were carried out to analyze differences in each preference. Participants rated travel preferences for self-esteem and prestige (*t* = 4.15, *p* < 0.001), finding new challenges and emotions (*t* = 2.26, *p* = 0.025), circuit visits (*t* = 2.86, *p* = 0.005), and travel for cultural and historic reasons (*t* = 2.24, *p* = 0.027) as less important now than in the year before the SARS-CoV-2 pandemic. They rated preferences for in-country travels (*t* = −3.49, *p* = 0.001), relief (*t* = −2.06, *p* = 0.041), and quality of facilities (*t* = −2.89, *p* = 0.005) as more important. No significant differences were found regarding knowledge and social interaction preferences (*p* > 0.05).

### 3.4. Factors Associated with COVID-19-Related Travel Behavior (Research Question 2)

In bivariate analyses, variables that had associations at *p* < 0.05 with travel intention included fun-seeking personality traits (*p* < 0.001) and preferences for stimulation and fun (*r* = 0.33, *p* = 0.001). Variables that had associations at *p* < 0.05 with travel avoidance included life impact (*p* = 0.028), media exposure (*p* = 0.012), fear of travel (*p* < 0.001), severity of contacting COVID-19 at destination (*p* < 0.001), risk of infection (*p* = 0.004), problem-solving coping (*p* = 0.002), and risk of transmitting the disease to loved ones after travel (*p* = 0.001). Higher levels of travel avoidance were also associated with higher preferences for safety (*r* = 0.32, *p* = 0.001) and relief (*r* = 0.32, *p* = 0.001). Variables that had associations at *p* < 0.01 with cautious travel included life impact (*p* = 0.001), media exposure (*p* < 0.001), fear of travel (*p* < 0.001), severity of contacting COVID-19 at destination (*p* < 0.001), risk of infection (*p* = 0.008), problem-solving coping (*p* < 0.001), and risk of transmitting the disease to loved ones after travel (*p* = 0.001). In addition to travel avoidance, individuals with high levels of cautious travel had higher levels of self-efficacy in controlling the COVID-19 infection (*p* < 0.001) and social-supported emotional coping (*p* < 0.001). Similarly to travel avoidance, individuals with higher levels of cautious travel had higher preferences for safety (*r* = 0.46, *p* = 0.001) and relief (*r* = 0.30, *p* = 0.001) (see Table 2).

To investigate which predictors uniquely explained the variation in the travel behavior across demographic variables, all significant continuous predictors were entered into a stepwise multivariate regression model for each travel behavior (travel intention, travel avoidance, and cautious travel). In the first step, gender, income, education, employment status, and age were entered. In the next step, the significant continuous predictors for each travel behavior were entered. Multivariate regression analyses for travel behaviors as outcome variables can be found in Table 3.

Adding the significant continuous predictors (risk, cognitive, personality, and coping variables) to the model explained 20% of the variance in intention to travel (F(2, 102) = 7.88, *p* < 0.001), 46% of the variance in travel avoidance (F(8, 96) = 9.51, *p* < 0.001), and 61% of the variance in cautious travel (F(11, 93) = 8.90, *p* < 0.001) after removing the travel restrictions.

Intention to travel was predicted by fun-seeking personality traits (*β* = −0.66, *t* = 3.87, *p* < 0.001), whereas problem solving and demographic variables did not significantly predict higher levels of intention to travel (*p* > 0.05). Participants’ intention to travel increased by 0.66 points for each point on the fun-seeking scale. Travel avoidance was predicted by high rates of fear of travel (*β* = 0.65, *t* = 5.89, *p* < 0.001), whereas demographic variables, severity of contracting COVID-19 at the destination, risk of infection and transmitting SARS-CoV-2 upon returning from travel, and coping did not significantly predict higher levels of travel avoidance (*p* > 0.05).

Cautious travel was predicted by fear of travel (*β* = 0.21, *t* = 2.78, *p* = 0.007) along with high levels of self-efficacy(Self-efficacy is inversely scored, lower score indicating high self-efficacy.) (*β* = −0.40, *t* = −3.43, *p* = 0.001), problem-solving coping (*β* = 0.44, *t* = 4.49, *p* < 0.001), and age (*β* = 0.38, *t* = 2.69, *p* = 0.008). Severity of contracting COVID-19 at the destination, risk of transmitting SARS-CoV-2 upon returning from travel, and media exposure did not significantly predict higher levels of cautious travel in the second step (*p* > 0.05). Table 3 provides the standardized regression coefficients of the predictors in the stepwise regression model.

### 3.5. Factors Associated with COVID-19: Preferences for Relief and Relaxation, Safety, Stimulation, and Prestige in Travel (Research Question 3)

We asked participants how important it was for them to impress others (prestige and self-esteem), to escape from stress and relax (relief), to ensure safety and protection measures (safety), and to have entertaining and stimulating experiences (stimulation) when planning to travel in the present.

Similarly to the analyses regarding travel behavior, variables that had associations at *p* < 0.01 with prestige included drive (*r* = −0.30, *p* = 0.001), fun-seeking (*r* = −0.22, *p* = 0.016), self-emotional coping (*r* = 0.28, *p* = 0.002), social-supported emotional coping (*r* = 0.28, *p* = 0.003), and collective coping (*r* = 0.30, *p* = 0.001). Variables that had associations at *p* < 0.01 with relief included drive (*r* = −0.19, *p* = 0.047), behavioral inhibition (*r* = −0.21, *p* = 0.027), life impact (*r* = 0.34, *p* < 0.001), risk of infection (*r* = 0.30, *p* = 0.001), risk of transmitting the disease to loved ones after travel (*r* = 0.23, *p* = 0.013), severity of contracting COVID-19 at the destination (*r* = 0.43, *p* < 0.001), coping (*p* < 0.05), and fear of travel (*r* = 0.36, *p* < 0.001). Relief was also related to travel avoidance (*r* = 0.26, *p* = 0.005) and cautious travel (*r* = 0.30, *p* = 0.001). Variables that had associations at *p* < 0.01 with safety included media exposure (*r* = 0.19, *p* = 0.046), risk of infection (*r* = 0.39, *p* < 0.001), risk of transmitting the disease to loved ones after travel (*r* = 0.28, *p* = 0.002), severity of contracting COVID-19 at the destination (*r* = 0.30, *p* = 0.001), coping (*p* < 0.05 except disengagement), and fear of travel (*r* = 0.35, *p* < 0.001). Safety was also related to travel avoidance (*r* = 0.32, *p* = 0.005) and cautious travel (*r* = 0.46, *p* < 0.001).

Variables that had associations at *p* > 0.01 with stimulation preferences included drive (*r* = −0.26, *p* = 0.005), fun-seeking (*r* = −0.37, *p* < 0.001), behavioral inhibition (*r* = 0.26, *p* < 0.005), self-emotional coping (*r* = 0.25, *p* = 0.007), collective coping (*r* = 0.26, *p* = 0.005), and intention to travel (*r* = 0.33, *p* < 0.001).

To investigate which predictors uniquely explained the variation in travel preferences, all significant continuous predictors were entered into a simultaneous multivariate regression model for each preference as a separate dependent variable. This model explained 41% of the variance in relief preferences (*F*(12, 97) = 5.68, *p* < 0.001), 32% of the variance in safety preferences (*F*(9, 100) = 5.33, *p* < 0.001), 26% of the variance in preferences for stimulation (*F*(5, 104) = 7.53, *p* < 0.001), and 19% of the variance in preferences for prestige (*F*(5, 104) = 5.12, *p* < 0.001) after removing the travel restrictions.

Prestige preferences were significantly (marginally) predicted by drive (*β* = −0.38, *p* = 0.051), whereas stimulation preferences were significantly predicted by fun-seeking (*β* = −0.71, *p* = 0.006), and inversely by behavioral inhibition (*β* = 0.60, *p* = 0.008) and self-focused emotional coping (*β* = 0.29, *p* = 0.040).

Relief and relaxation preferences were significantly predicted by the severity of contacting COVID-19 at the destination (*β* = 0.26, *p* = 0.023) and self-focused emotional coping (*β* = 0.32, *p* = 0.013), whereas drive, behavioral inhibition, of for transmitting the disease to loved ones after travel, collective coping, and social-supported emotional coping did not predict relief preferences.

Safety preferences were significantly predicted only by the perceived risk of infection at the destination (*β* = 0.29, *p* = 0.019) and self-focused emotional coping (*β* = 0.47, *p* = 0.001), whereas risk of transmitting the disease to loved ones after travel, severity of contracting COVID-19 at the destination, fear of travel, problem-solving coping, collective coping, and social-supported emotional coping did not predict safety preferences.

## 4. Discussion

The present study investigated predictors of, and changes in, travel behavior via an online survey study. Based on the literature, we expected (1) that individuals would prefer home destinations over foreign destinations, and (2) that SARS-CoV-2 exposure, along with cognitive, personality, and affective factors (e.g., direct and media exposure, perceptions of risk to the self and others, efficacy, coping, and fear of travel) would predict travel behavior and travel preferences during the COVID-19 pandemic.

We found that participants reported greater preference for traveling in their home country. Regarding the reported traveling, participants reported less travel during the pandemic period compared to the pre-pandemic period in terms of traveling abroad. Regarding traveling in the home country, we found a different pattern; as expected, participants reported that they traveled less often in groups in their home country; however, more participants reported that they traveled 1–3 times during the pandemic than in the year before. Thus, we found that more individuals engaged in “low travels” in their country and surroundings during the pandemic than before. For “high travelers”, we found a different pattern; this pattern suggests that during pandemics, although more people travel, “high travelers” travel less often. It is possible that participants who used to go on holidays abroad shifted to locations in their own country. This finding is consistent with an increase in travel at home and by personal car during the pandemic [6].

We also found that behavioral intention was uniquely predicted by the personality trait of fun-seeking, regardless of demographic variables. Intention to travel was also found to increase with age and education. We did not find any relation between BIS and travel avoidance or cautious travel. Both travel avoidance and cautious travel were predicted by fear of travel. While travel avoidance was predicted by fear of travel alone, fear of travel, problem-solving coping, self-efficacy in controlling infection with SARS-CoV-2, and risk of contracting SARS-CoV-2 uniquely explained cautious travel.

From the different psychological vulnerability factors, we found only fun-seeking to be a significant predictor of travel intention in the stepwise regression model, regardless of demographic variables. Additionally, age and education uniquely predicted intention to travel. This result suggests that older and more educated participants (who probably travel more) had higher levels of intention to travel. It is interesting that fun-seeking explained additional variance beyond the variance explained by problem solving. This indicates that fun-seeking is a unique component in explaining travel intention. This is a new finding, given that neuropsychological traits of personality have not (to our knowledge) been investigated in relation to intention to travel during the COVID-19 pandemic. Previous studies investigating the role of personality in travel, in relation to perceptions of risks regarding wildfires, found that novelty-seeking and sensation-seeking were not related to courageous behavior [50]. Given that fun-seeking may represent openness to new experiences, searching for new rewards in an impulsive manner and, notably, low constraint [68], it may be a stronger predictor of the intention to travel when the risk of COVID-19 is still present.

We found that fear of travel independently predicts travel avoidance and cautious travel after lifting the travel restrictions. This suggests that both factors independently explain variance in travel avoidance and cautious travel. Our results add to previous findings regarding the role of fear of travel in travel avoidance and cautious travel [43]. We did not find that media exposure was an independent factor in travel avoidance. This is in contrast with previous studies that found a direct relationship between regular and social media exposure and fear of COVID-19 [69].

We also found that cautious travel is uniquely predicted by fear of travel, but also by self-efficacy, problem-solving coping, and perceived risk of contracting SARS-CoV-2 infection. This result is consistent with previous findings of Zheng et al., who found that higher levels of coping and self-efficacy result in cautious travel behavior rather than travel avoidance [43]. Thus, it is possible that individuals who fear to travel due to COVID-19 will not avoid travel, but will shift to cautious travel when they perceive high risk of infection but also feel that they can efficiently control the infection and solve problems related to COVID-19. Furthermore, age also uniquely predicted cautious travel. This is consistent with a more general tendency for cautious behavior to increase with age [6].

Our study showed that participants considered preferences for prestige, self-esteem, and stimulation as being less important when they travel. Contrary to our prediction, participants rated safety and relief (marginally) as being similarly important preferences for travel both during COVID-19 and in the year before the COVID-19 pandemic. This may be due to high levels of safety and relief preferences in our sample. We found that different factors play distinct roles in predicting specific travel preferences after removing the restrictions. First, we found that self-focused emotional coping predicted preferences for relief (together with high levels of perceived severity) and safety (together with high levels of perceived risk of infection). These results place travel preferences among larger mechanisms of coping. It is possible that a preference for safety occurs as a protection motivation in individuals who try to calm themselves when they perceive higher risks in the context of thinking of traveling [43]. Similarly, a preference for relief may occur as a protection motivation in individuals who try to calm themselves when they perceive a higher severity of contracting COVID-19 at a travel destination. Interestingly, we found higher stimulation preferences during the pandemic in individuals with lower behavioral inhibition and higher fun-seeking. This is consistent with the threat and defense model of BIS/BAS, in which behavioral activation is the second phase of coping with adversity [70]. Thus, a decrease in behavioral inhibition may result in higher levels of behavioral activation and engagement of preferences for stimulation and enjoyment. Contrary to our expectation, we did not find behavioral inhibition to be a predictor of travel avoidance. However, behavioral inhibition was related to fear of travel, replicating the findings of Oniszczenko on BIS and fear of COVID-19 [51].

There are several factors that may influence this relationship. It may be possible that travel avoidance may be a decision based on more factors than just behavioral inhibition. For instance, travel avoidance decisions may be related to high probability of lockdown at the travel destination. Other factors may include the possibility for refunding if journeys are cancelled, general financial instability, or not knowing whether close relatives may get ill, resulting in travel cancelation.

Second, our measure of behavioral inhibition refers to general cognitive reactions to future aversive conditions. Travel avoidance is a coping mechanism that resolves aversive conflict. It is possible that fear of travel may be a better measure to understand behavioral inhibition during the pandemic than personality measures. It is possible that the scores of behavioral inhibition were inflated due to high levels of anxiety during the period of completion. Thus, individuals who do not have high levels of behavioral inhibition may endorse high-anxiety items on the behavioral inhibition scale, resulting in false positives. If this is the case, some participants scoring high on the BIS scale (due to high anxiety during pandemic) will not display high levels of travel avoidance.

Some suggestions for the management of travel in the post-pandemic period can be made based on our findings. One important suggestion follows the finding that cautious travel is explained by fear of travel along with higher levels of self-efficacy and problem-solving coping when participants perceive higher risk levels. It may be the case that measures that promote self-efficacy and problem solving will shift from travel avoidance to cautious travel in the face of risk perceptions. Thus, messages of control over methods to prevent infection and solutions for managing the severity of infection may foster self-efficacy and cautious travel rather than travel avoidance. This result confirms the findings of Zheng et al. [43], which suggest that successful coping (including problem solving) is linked with resilience, and promotes cautious travel.

The observed relationship between fear of travel and travel avoidance suggests that addressing fear of travel is important to reduce avoidance and stimulate travel. Our analysis implies that some factors—such as perceived severity of contracting COVID-19 at the destination, the risk of contracting COVID-19, and the risk of transmitting SARS-CoV-2 upon returning—may exacerbate fear of travel and result in avoidance of travel. Thus, they are the best candidates for reducing fear of travel. Clear communication about what to do when infected at the travel destination, how to prevent infection (increasing self-efficacy to control infection), and providing quality health and medical services at the travel destination may reduce fear of travel and travel avoidance. Moreover, communicating about measures of reducing the risk of transmitting the virus upon returning may result in decreases in fear of travel.

Intention to travel was predicted by fun-seeking; this has several implications. Individuals with high levels of fun-seeking also have the tendency to be impulsive, and have low compliance with rules; thus, extra caution for respecting measures should be considered. Since impulsivity may hamper rules such as wearing masks and social distancing, control measures based on environmental modifications should be preferred to those based on individual decisions. For instance, limiting the number of tourists at the destination or accessing facilities should be preferred over measures relying on tourist decisions.

Our current findings, however, have several limitations. First, they were limited by exclusive reliance on self-report questionnaires. It would be beneficial for future research to incorporate observation and neuropsychological measures for behavioral inhibition and fun-seeking subsystems. Moreover, the limited number of participants imposed limitations in interpreting the results. Additional studies should use larger samples to confirm our findings. Recruitment of participants occurred via social media tourism groups and by email. Thus, the sample was skewed toward young respondents. Furthermore, having more women than men in our sample limits the generalizability of our findings. Women are more risk-aversive and more avoidant than men [6]. Moreover it is possible that the relationship between personality and travel behavior is significant only for women and not for men. To verify this case, we ran a series of exploratory moderation analyses using Hayes process model 1 [71], using personality as a predictor, gender as a moderator, and travel behavior as the outcome variable. Our results showed no significant moderation by gender for the relationships between BIS and travel avoidance (R square increase due to interaction being 0.00, *p* = 0.7743), fun-seeking and travel intention (R square increase due to interaction being 0.00, *p* = 0.941), fear of travel and travel avoidance (R square increase due to interaction being 0.00, *p* = 0.948), or fear of travel and travel caution (R square increase due to interaction being 0.00, *p* = 0.594). Another limit is self-selection bias. We disseminated the survey via Facebook groups in the country, student groups, and by email. It is possible that the travel behavior in our sample differs from the travel behavior in the general population. While we found similar levels of cautious travel and intention to travel, we observed lower levels of travel avoidance and fear of travel than those observed in previous studies; however, this difference was expected. We collected data in the summer of 2021, after a relaxation period and low case reports in Romania. The study of Zenker et al. recruited participants in weeks 25–26 of 2020, when there was a spike of COVID-19 cases in the US [63]. Although these differences may be explained by the period of assessment (we assessed participants during a period of relaxation), they may also result from self-selection bias, and require cautious interpretation. Similarly, the personalities of our respondents may differ from the personalities of the target population. Because of self-selection bias, we expected that fun-seeking would be greater in our sample than in the population. Exploratory analysis showed that the means of the personality scales (fun-seeking, drive, reward responsiveness, and behavioral inhibition) were similar to the means obtained in other Romanian samples [72,73], as well as those in other, larger samples. Nevertheless, an important limitation comes from the fact that we investigated local residents’ travel behavior based on travel intentions and self-reported traveling. It is possible that there were differences between self-reports and actual travel, which may have been influenced by factors at the moment of completion of the survey. Further studies should include both self-report and objective data in their analyses.

## 5. Conclusions

In conclusion, in this online study, we found that respondents had a high intention to travel, yet an important percentage still expressed high levels of travel avoidance. Furthermore, fear of travel was related to travel avoidance. Since many individuals who display high levels of intention to travel after removing restrictions have higher levels of fun-seeking, infection control measures based on environmental modifications (e.g., social distancing based on limiting the distance by means of physical obstacles) at travel destinations rather than control measures based on personal agency (e.g., social distancing based on individuals keeping the distance) should be preferred.

## Figures and Tables

**Table 1:** Descriptive statistics for the demographic data.

	Frequency	%
Age (years)		
0–18	-	-
19–30	51	46.4
31–45	47	42.7
45–60	11	10
60+	-	-
Gender (F/M, n, %)		
Male	25	31.8
Female	75	68.2
Education (years of study)		
High school	27	24.5
Undergraduate	41	37.3
Postgraduate	41	37.3
Income		
EUR < 250	34	30.9
EUR 250–450	17	15.5
EUR 450–900	34	30.9
EUR > 900	25	22.7
Employment		
Employed	64	52.8
Not employed	4	3.6
Not employed due to pandemic	3	2.7
Student	33	30
Retired	1	0.9
Self-employed	5	4.5
Being infected with coronavirus	23	20.9
Having family, neighbors, or close friends infected with coronavirus	86	78.2
Vaccinated against SARS-CoV-2 or scheduled for vaccination	50	45.5
Planned to travel in this time period	78	71.8
Exposure to COVID-19 for the planned travel		
I do not know about the existence of COVID-19 in the destination country	15	13.6
COVID-19 is present in the destination country	9	44.5
COVID-19 is present in the destination city	19	17.3
COVID-19 is present in the destination area	22	20
Persons you know have been infected in the destination you visit	2	1.8
Close persons have been infected in the destination you visit	3	2.7

**Table 2:** Correlation coefficients between travel behaviors and risk, media exposure, severity of disease, coping, and personality variables.

	1	2	3	4	5	6	7	8	9	10	11	12	13	14
1.Intention to travel	1	-	-	-	-	-	-	-	-	-	-	-	-	-
2.Travel avoidance	−0.18	1	-	-	-	-	-	-	-	-	-	-	-	-
3.Cautious travel	0.07	0.42 **	1	-	-	-	-	-	-	-	-	-	-	-
4.Fun-seeking	−0.34 **	0.17	−0.00	1	-	-	-	-	-	-	-	-	-	-
5.BIS	0.12	−0.15	−0.02	−0.10	1	-	-	-	-	-	-	-	-	-
6.FOT	−0.15	0.61 **	0.41 **	0.23 *	−0.34 **	1	-	-	-	-	-	-	-	-
7.Life impact	0.00	0.20 *	0.30 **	−0.24 *	−0.10	0.17	1	-	-	-	-	-	-	-
8.Media exposure	−0.04	0.23 *	0.32 **	0.01	−0.04	0.20 *	0.35 **	1	-	-	-	-	-	-
9.Severity of infection	−0.15	0.43 **	0.35 **	0.09	−0.31 **	0.50 **	0.36 **	0.25 **	1	-	-	-	-	-
10.Risk of infection	0.06	0.27 **	0.42 **	−0.02 *	−0.07	0.28 **	0.24 **	0.20 *	0.43 **	1	-	-	-	-
11.Risk of transmitting	−0.09	0.30 **	0.25 **	0.01	−0.31 **	0.37 **	0.27 **	0.26**	0.57**	0.53 **	1	-	-	-
12. Self-efficacy	−0.14	0.00	−0.32 **	0.04	−0.20*	0.01	−0.01	−0.01	0.10	−0.11	0.13	1	-	-
13.Problem-solving coping	0.08	0.28 **	0.56 **	−0.02	0.06	0.27 **	0.23 *	0.45 **	0.22 *	0.14	0.15	−0.06	1	-
14.Emotion coping	0.04	0.16	0.32 **	−0.05	−0.02	0.34 **	0.29 **	0.29 **	0.21 *	0.20 *	0.18	0.06	0.58 **	1

Note: FOT: fear of travel; BIS: behavoral inhibition system; * *p* < 0.05 (2-tailed); ** *p* < 0.001 (2-tailed).

**Table 3:** Regression coefficients of predictors of cautious travel, travel avoidance, and intention to travel.

Cautious Travel				Travel Avoidance				Intention to Travel			
Predictor	*β*	*t*	*p*	Predictor	*β*	*t*	*p*	Predictor	*β*	*t*	*p*
Block 1 R square = 0.21, *p* < 0.01				Block 1 R square = 0.03, *p* = 0.64				Block 1 R square = 0.08, *p* = 0.09			
Gender	0.00	0.02	0.984	Gender	0.13	0.49	0.620	Gender	0.08	0.44	0.656
Age	0.22	1.29	0.197	Age	0.39	1.66	0.099	Age	−0.21	−1.25	0.212
Education	0.51	4.08	0.000 **	Education	0.06	0.35	0.723	Education	0.28	2.22	0.028 *
Employment	−0.01	−0.24	0.806	Employment	0.02	0.21	0.834	Employment	0.11	1.73	0.086
Income	−0.06	−0.57	0.570	Income	−0.15	−0.94	0.345	Income	0.14	1.26	0.209
Block 2 R square = 0.61, *p* < 0.01				Block 2 R square = 0.46, *p* < 0.01				Block 2 R square = 0.20, *p* < 0.01			
Gender	0.01	0.09	0.921	Gender	−0.02	−0.10	0.920	Gender	0.04	0.22	0.824
Age	0.38	2.69	0.008*	Age	0.34	1.66	0.099	Age	−0.21	−1.31	0.192
Education	0.12	1.09	0.277	Education	−0.27	−1.83	0.069	Education	0.26	2.19	0.030 *
Employment	0.00	−.170	0.865	Employment	0.08	1.06	0.289	Employment	0.13	2.04	0.044 *
Income	−0.09	−1.04	0.298	Income	−0.07	−0.61	0.538	Income	0.16	1.52	0.130
Media	−0.11	−1.06	0.290	Media	0.02	0.12	0.899	Fun-seeking	−0.66	−3.87	0.000 **
General impact	0.12	1.17	0.241	General impact	0.11	0.82	0.411	Problem solving	0.06	0.66	0.508
Anticipated exposure	0.02	0.40	0.686	Risk of contracting COVID-19	0.19	1.37	0.174				
Risk of contracting COVID-19	0.30	3.05	0.003 *	Severity of contracting COVID-19	0.13	0.93	0.350				
Severity of contracting COVID-19	0.00	0.03	0.973	Risk of transmision -	−0.06	−0.53	0.597				
Risk of transmision	−0.03	−0.47	0.636	Problem solving	0.17	1.36	0.177				
Problem-solving	0.44	4.49	0.000 **	Self-focused coping	−0.06	−0.41	0.679				
Self-efficacy *	−0.40	−3.47	0.001 **	Fear of Travel	0.65	5.89	0.000 **				
Self-focused coping	0.15	1.37	0.172								
Social-focused coping	−0.13	−1.36	0.174								
Fear of Travel	0.21	2.78	0.007 *								

*Note*: *β* = unstandardized regression coefficients; * p < 0.05 (2-tailed), ** *p* < 0.01 (2-tailed). Self-efficacy is inversely scored, with lower scores indicating higher self-efficacy.

## Data Availability

Not applicable.

## References

[1] Ilies A., Grama V. (2010). The External Western Balkan Border of the European Union and Its Borderland: Premises for Building Functional Transborder Territorial Systems. Ann. Anal. Za Istrske. Mediter. Studije. Ser. Hist. Et Sociol..

[2] Ilies D.C., Onet A., Marcu F.M., Gaceu O.R., Timar A., Baias S., Ilies A., Herman G.V., Costea M., Tepelea M. (2018). Investigations on Air Quality in The Historic Wooden Church in Oradea City, Romania. Environ. Eng. Manag. J..

[3] Wendt J.A., Grama V., Ilieş G., Mikhaylov A.S., Borza S.G., Herman G.V., Bógdał-Brzezińska A. (2021). Transport Infrastructure and Political Factors as Determinants of Tourism Development in the Cross-Border Region of Bihor and Maramureş. A Comparative Analysis. Sustainability.

[4] International Tourism Growth Continues to Outpace the Global Economy. https://www.unwto.org/international-tourism-growth-continues-to-outpace-the-economy.

[5] Giddy J.K., Rogerson J.M. (2021). Nature-Based Tourism Enterprise Adaptive Responses to Covid-19 in South Africa. GeoJ. Tour. Geosites.

[6] Abdullah M., Dias C., Muley D., Shahin M. (2020). Exploring the impacts of COVID-19 on travel behavior and mode preferences. Transp. Res. Interdiscip Perspect..

[7] Jones C., Philippon T., Venkateswaran V. (2020). Optimal Mitigation Policies in a Pandemic: Social Distancing and Working from Home.

[8] Yilmazkuday H. (2020). COVID-19 Deaths and Inter-County Travel: Daily Evidencefrom the US. Transp. Res. Interdiscip. Perspect..

[9] Awad-Núñez S., Julio R., Gomez J., Moya-Gómez B., Sastre González S.J. (2021). Post-COVID-19 travel behaviour patterns: Impact on the willingness to pay of user of public transport and shared mobility services in Spain. Eur. Transp. Res. Rev..

[10] Herman G.V., Banto N., Caciora T., Ungureanu M., Furdui S., Grama V., Buhaș R., Buhaș S. (2020). Tourism in Bihor County, Romania. Trends and Prospects. Folia Geographica.

[11] Ilies A., Hurley P.D., Ilies D.C., Baias S. (2017). Tourist Animation—A Chance for Adding Value to Traditional Heritage: Case Studies in The Land of Maramures (Romania). Rev. De Etnogr. Si Folc. J. Ethnogr. Folk..

[12] Ilies D.C., Onet A., Grigore H., Liliana I., Alexandru I., Ligia B., Ovidiu G., Florin M., Stefan B., Tudor C. (2019). Exploring the Indoor Environment of Heritage Buildings and Its Role in the Conservation of Valuable Objects. Environ. Eng. Manag. J..

[13] Manhas P.S., Singh R., Fodor G., Berghauer S., Mir M.A., David L.D. (2021). Examination of Impact of Responsible Tourism Practices on Quality of Life of Destination Communities. GeoJ. Tour. Geosites.

[14] Morar C., Berman L., Unkart S., Erdal S. (2021). Sustainable Brownfields Redevelopment in the European Union: An Overview of Policy and Funding Frameworks. J. Environ. Health.

[15] Morar C., Lukić T., Basarin B., Valjarević A., Vujičić M., Niemets L., Telebienieva I., Nagy G. (2021). Shaping Sustainable Urban Environments by addressing the Hydro-Meteorological Factors in the Landslide Occurrence: Ciuperca Hill (Oradea, Romania). Int. J. Environ. Res. Public Health.

[16] Morar C., Nagy G., Boros L., Gozner M., Niemets L., Sehida K. (2021). Heritage, Culture and Regeneration of the former Military Areas in the city of Oradea (Romania). Archit. Urban..

[17] Morar C., Nagy G., Dulca M., Boros L., Sehida K. (2019). Aspects Regarding the Military Cultural-Historical Heritage in the City of Oradea, Romania. Ann. Ann. Istrian Mediterr. Stud. Ser. Hist. Et Sociol..

[18] Burns A.J., Posey C., Roberts T.L., Benjamin Lowry P. (2017). Examining the relationship of organizational insiders’ psychological capital with information security threat and coping appraisals. Computers Hum. Behav..

[19] Chinazzi M., Davis J.T., Ajelli M., Gioannini C., Litvinova M., Merler S., Piontti A.P., Mu K., Rossi L., Sun K. (2020). The effect of travel restrictions on the spread of the 2019 novel coronavirus (COVID-19) outbreak. Science.

[20] Kraemer M.U., Yang C.-H., Gutierrez B., Wu C.-H., Klein B., Pigott D.M., Du Plessis L., Faria N.R., Li R., Hanage W.P. (2020). The effect of human mobility and control measures on the COVID-19 epidemic in China. Science.

[21] Linka K., Peirlinck M., Sahli Costabal F., Kuhl E. (2020). Outbreak dynamics of COVID-19 in Europe and the effect of travel restrictions. Comput. Methods Biomech. Biomed. Eng..

[22] Sui W., Prapavessis H. COVID-19 has Created More Cyclists: How Cities Can Keep Them on Their Bikes. The Conversation. https://theconversation.com/covid-19-has-created-more-cyclists-how-cities-can-keep-them-on-their-bikes-137545.

[23] TREK (2020). Data indicates Americans are Turning to Cycling for Mental and Physical Health as Well as an Alternate Means of Essential Transportation during COVID-19.

[24] Guse C. NYC Subway Ridership Hits Highest Point since March as New Yorkers Adjust to Coronavirus Pandemic. https://www.nydailynews.com/coronavirus/ny-coronavirus-subway-ridership-uptick-20200520-vowavu6aoffgzjbrwwwnkibe5q-story.html.

[25] Teixeira J.F., Lopes M. (2020). The link between bike sharing and subway use during the COVID-19 pandemic: The case-study of New York’s Citi Bike. Transp. Res. Interdiscip. Perspect..

[26] Vyas Padmanabhan V., Penmetsa P., Li X., Dhondia F., Dhondia S., Parrish A. (2021). COVID-19 effects on shared-biking in New York, Boston, and Chicago. Transp. Res. Interdiscip. Perspect..

[27] Yan Q., Tang S., Xiao Y. (2018). Impact of individual behaviour change on the spread of emerging infectious diseases. Stat. Med..

[28] Perić G., Dramićanin S., Conić M. (2021). The impact of Serbian tourists’ risk perception on their travel intentions during the COVID-19 pandemic. Eur. J. Tour. Res..

[29] Yang E.C.L., Nair V. (2014). Tourism at risk: A review of risk and perceived risk in tourism. Asia-Pac. J. Innov. Hosp. Tour..

[30] Pizam A., Jeong G., Reichel A., Van Boemmel H., Lusson J., Steynberg L., State-Costache O., Volo S., Kroesbacher C., Hucerova J. (2004). The relationship between risk-taking, sensation-seeking, and the tourist behavior of young adults: A cross-cultural study. J. Travel Res..

[31] Gibson H., Yiannakis A. (2002). Tourist roles: Needs and the lifecourse. Ann. Tour. Res..

[32] Lepp A., Gibson H. (2003). Tourist roles, perceived risk and international tourism. Annals Tour. Res..

[33] Kozak M., Crotts J.C., Law R. (2007). The impact of the perception of risk on international travellers. Int. J. Tour. Res..

[34] Lepp A., Gibson H. (2008). Sensation seeking and tourism: Tourist role, perception of risk and destination choice. Tour. Manag..

[35] Neuburger L., Egger R. (2020). Travel risk perception and travel behaviour during the COVID-19 pandemic 2020: A case study of the DACH region. Curr. Issues Tour..

[36] Bae S.Y., Chang P.J. (2020). The effect of coronavirus disease-19 (COVID-19) risk perception on behavioural intention towards ‘untact’ tourism in South Korea during the first wave of the pandemic (March 2020). Curr. Issues Tour..

[37] Kourgiantakis M., Apostolakis A., Dimou I. (2020). COVID-19 and holiday intentions: The case of Crete, Greece. Anatolia.

[38] Ivanova M., Ivanov I.K., Ivanov S. (2020). Travel behaviour after the pandemic: The case of Bulgaria. Anatolia.

[39] Bratić M., Radivojević A., Stojiljković N., Simović O., Juvan E., Lesjak M., Podovšovnik E. (2021). Should I Stay or Should I Go? Tourists’ COVID-19 Risk Perception and Vacation Behavior Shift. Sustainability.

[40] Niemets L., Kostrikov S., Kandyba Y., Dobrovolskaya N., Kobylin P., Kucheriava H., Soliman K.S. Features and problematic issues of the tourism development in Ukraine due to the coronavirus Covid-19 pandemic. Proceedings of the 36th International Business Information Management Association Conference (IBIMA).

[41] Strong P. (1990). Epidemic psychology: A model. Soc. Health Illn..

[42] Ahorsu D.K., Lin C.Y., Imani V., Saffari M., Griffiths M.D., Pakpour A.H. (2020). The Fear of COVID-19 Scale: Development and Initial Validation. Int. J. Ment. Health Addict..

[43] Zheng D., Luo Q., Ritchie B.W. (2021). Afraid to travel after COVID-19? Self-protection, coping and resilience against pandemic ‘travel fear’. Tour. Manag..

[44] Fennell D.A. (2017). Towards a model of travel fear. Ann. Tour. Res..

[45] Somma A., Gialdi G., Krueger R.F., Markon K.E., Frau C., Lovallo S., Fossati A. (2020). Dysfunctional personality features, non-scientifically supported causal beliefs, and emotional problems during the first month of the COVID-19 pandemic in Italy. Personal. Individ. Differ..

[46] Aschwanden D., Strickhouser J.E., Sesker A.A., Lee J.H., Luchetti M., Stephan Y., Sutin A.R., Terracciano A. (2020). Psychological and Behavioural Responses to Coronavirus Disease 2019: The Role of Personality. Eur. J. Personal..

[47] Moccia L., Janiri D., Pepe M., Dattoli L., Molinaro M., De Martin V., Chieffo D., Janiri L., Fiorillo A., Sani G. (2020). Affective temperament, attachment style, and the psychological impact of the COVID-19 outbreak: An early report on the Italian general population. Brain Behav. Immun..

[48] Fernández R.S., Crivelli L., Guimet N.M., Allegri R.F., Pedreira M.E. (2020). Psychological distress associated with COVID-19 quarantine: Latent profile analysis, outcome prediction and mediation analysis. J. Affect. Disord..

[49] Prentice C., Zeidan S., Wang X. (2020). Personality, trait EI and coping with COVID 19 measures. Int. J. Disaster Risk Reduct..

[50] Kovačić S., Margarint M.C., Ionce R., Miljković Đ. (2020). What are the Factors Affecting Tourist Behavior Based on the Perception of Risk? Romanian and Serbian Tourists’ Perspective in the Aftermath of the recent Floods and Wildfires in Greece. Sustainability.

[51] Oniszczenko W. (2021). The association between BIS/BAS and fear of COVID-19 infection among women. Curr. Issue Personal. Psychol..

[52] Carver C.S., White T.L. (1994). Behavioral inhibition, behavioral activation, and affective responses to impending reward and punishment: The BIS/ BAS scales. J. Person. Soc. Psychol..

[53] Gray J.A. (1982). The Neuropsychology of Anxiety: An Enquiry into the Functions of the Septo-Hippocampal System of the Septo-Hippocampal system.

[54] Gray J.A. (1990). Brain systems that mediate both emotion and cognition. Cognit. Emot..

[55] Chew E.Y.T., Jahari S.A. (2014). Destination image as a mediator between perceived risks and revisit intention: A case of post-disaster Japan. Tour. Manag..

[56] Biran A., Liu W., Li G., Eichhorn V. (2014). Consuming post-disaster destinations: The case of Sichuan, China. Ann. Tour. Res..

[57] Niemets L., Kandyba Y., Kobylin P., Kostrikov S., Dobrovolskaya N., Telebienieva I., Soliman K.S. (2021). Integral Assessment of Ethnic Tourism in Ukraine: Resource Provision and Regional Features. Innovation Management and Information Technology Impact on Global Economy in the Era of Pandemic, Proceedings of the 37th International Business Information Management Association Conference (IBIMA), Cordoba, Spain, 1–2 April 2021.

[58] Niemets L., Telebienieva I., Scryl I., Kucheriava H., Kornus A., Kliuchko L., Soliman K.S. (2021). Current State of Child and Youth Tourism Development in Ukraine (Case Study of Kharkiv Region). Innovation Management and Information Technology Impact on Global Economy in the Era of Pandemic, Proceedings of the 37th International Business Information Management Association Conference (IBIMA), Cordoba, Spain, 1–2 April 2021.

[59] Aghdam E.S., Kheirollahi A., Niemets L.M. (2020). Perspectives of the Tourism Development in Terms of Water Crisis (Case Study of Iran). Visnyk V.N. Karazin Kharkiv Natl. Univ. Ser. Geol. Geogr. Ecol..

[60] Niemets L., Telebienieva I., Sehida K., Krainiukov O., Kucheriava H., Pohrebskyi T., Soliman K.S. (2019). Features of pilgrimage tourism in Ukraine (a case study of Uman). Vision 2025: Education Excellence and Management of Innovations Through Sustainable Economic Competitive Advantage, Proceedings of the 34nd International Business Information Management Association Conference (IBIMA), Madrid, Spain, 13–14 November 2019.

[61] Niemets L., Sehida K., Lohvinova M., Kraynukov O., Kliuchko L., Soliman K.S. (2018). Rural Tourism in Ukraine: Peculiarities and Trends of Development. Sustainable Economic Development and Application of Innovation Management from Regional expansion to Global Growth, Proceedings of the 32nd International Business Information Management Association Conference (IBIMA), Seville, Spain, 15–16 November 2018.

[62] Georgiou N., Delfabbro P., Balzan R. (2020). COVID-19-related conspiracy beliefs and their relationship with perceived stress and pre-existing conspiracy beliefs. Pers. Individ. Differ..

[63] Zenker S., Braun E., Gyimóthy S. (2021). Too afraid to travel? Development of a pandemic (COVID-19) anxiety travel scale (PATS). Tour. Manag..

[64] Carver C.S. (1997). You want to measure coping but your protocol’too long: Consider the brief cope. Int. J. Behav. Med..

[65] Cann A., Calhoun L.G., Tedeschi R.G., Taku K., Vishnevsky T., Triplett K.N., Danhauer S.C. (2010). A short form of the Posttraumatic Growth Inventory. Anxiety Stress Coping.

[66] Workman M., Bommer W.H., Straub D. (2008). Security lapses and the omission of information security measures: A threat control model and empirical test. Comput. Hum. Behav..

[67] Lee C.-K., Song H.-J., Bendle L.J., Kim M.-J., Han H. (2012). The impact of nonpharmaceutical interventions for 2009 H1N1 influenza on travel intentions: A model of goal-directed behavior. Tour. Manag..

[68] Segarra P., Poy R., Lopez R., Molto J. (2014). Characterizing Carver and White’s BIS/BAS subscales using the Five Factor Model of personality. Pers. Individ. Differ..

[69] Mertens G., Gerritsen L., Duijndam S., Salemink E., Engelhard I.M. (2020). Fear of the coronavirus (COVID-19): Predictors in an online study conducted in March 2020. J. Anxiety Disord..

[70] Jonas E., McGregor I., Klackl J., Agroskin D., Fritsche I., Holbrook C., Nash K., Proulx T., Quirin M., Olson J.M., Zanna M.P. (2014). Threat and defense: From anxiety to approach. Advances in Experimental Social Psychology.

[71] Hayes A.F. (2013). Introduction to Mediation, Moderation, and Conditional Process Analysis: A Regression-Based Approach.

[72] Miu A.C., Bîlc M.I., Bunea I., Szentágotai-Tătar A. (2017). Childhood trauma and sensitivity to reward and punishment: Implications for depressive and anxiety symptoms. Pers. Individ. Differ..

[73] Tiba A.I. (2005). Demanding brain: Between should and shouldn’t. J. Cog. Behav. Psych..

